# Dynamic Cycle of Low Back Pain: A 17-Year, Population-Based Study Analyzing the National Health Insurance Service Data in South Korea

**DOI:** 10.3390/medicina61050782

**Published:** 2025-04-23

**Authors:** Mi-Ran Goo, Deok-Hoon Jun, Do-Youn Lee

**Affiliations:** 1 Department of Physical Therapy, Kyungwoon University, Gumi-si 39160, Republic of Korea; m.goo@ikw.ac.kr; 2 Department of Physical Therapy, Daegu University, Gyeongsan-si 38453, Republic of Korea; hoon.j@daegu.ac.kr; 3 College of General Education, Kookmin University, Seoul 02707, Republic of Korea

**Keywords:** episode patterns, recurrence, musculoskeletal disorders, hospital record, pain clinics, admissions, outpatients

## Abstract

*Background and Objectives*: Low back pain (LBP) is a highly prevalent musculoskeletal condition that frequently recurs, leading to increased healthcare utilization and socioeconomic burden. While short-term management strategies are well-documented, long-term recurrence patterns remain insufficiently studied. This study aims to describe the long-term recurrence patterns and healthcare utilization associated with LBP in a nationwide cohort over a 17-year period. *Materials and Methods*: This descriptive, retrospective longitudinal cohort study utilized data from the Korean National Health Insurance Service (NHIS) database (2002–2018). We included 3,086,665 patients who sought medical care for LBP (ICD-10 code M54.5) at least once in 2010. Patients with a history of disability rating assessments were excluded. The primary outcomes included the number of LBP episodes, episode duration, recurrence patterns, and changes in healthcare utilization. We assessed the number of healthcare visits per episode and the interval between episodes over time. *Results*: Among the study population, 79.4% experienced recurrent LBP, with an average of 5.0 ± 4.9 episodes per patient. Recurrence rates increased with each episode. In addition, episode duration lengthened, and intervals between episodes shortened. Healthcare utilization also increased, with patients requiring more visits per episode over time. The demographic and socioeconomic characteristics of the LBP patients in our sample were also described. *Conclusions*: In this population-based sample, LBP follows a progressive course, with increasing episode frequency, prolonged duration, and escalating healthcare utilization over time. These findings highlight the need for early intensive management and long-term follow-up strategies to mitigate the growing burden of recurrent LBP on individuals and healthcare systems.

## 1. Introduction

Chronic lower back discomfort is a significant global public health issue, affecting individuals across diverse demographic and socioeconomic groups [1,2]. Low back pain (LBP) is one of the most frequent reasons for patient visits in primary care, highlighting its substantial influence on healthcare resource utilization [3]. The widespread prevalence of this condition and its profound implications for individuals and healthcare systems have been extensively documented. LBP can result from a variety of underlying causes, including mechanical disorders such as muscle or ligament strain, intervertebral disc degeneration or herniation, and spinal stenosis [4]. However, non-specific LBP remains the most reported type [5,6]. Studies indicate that annual prevalence rates vary significantly across regions worldwide [2,7]. The influence of LBP extends beyond individual health, imposing considerable societal and economic burdens [8,9,10]. These burdens include direct healthcare costs and, more critically, reduced work productivity and functional capacity [11,12]. The working population is particularly affected, as low back-related disabilities significantly contribute to both short-term and long-term work absenteeism. Recent studies have suggested that low back pain may also be influenced by lifestyle-related factors, further supporting its classification as a lifestyle disease [13,14].

Understanding the episodic nature of LBP is crucial for assessing its effect on workforce productivity and societal burden. Research indicates that LBP often follows a recurrent pattern, with episodes typically reappearing within 1–3 years [15,16]. On average, 62% of patients with LBP report ongoing pain after 12 months, and annual recurrence rates range from 40 to 70% [17,18]. Furthermore, workers who sustain LBP-related injuries are at a higher risk of experiencing recurrent episodes within a year, a likelihood further heightened by age-related factors [19,20]. Short-term studies may inherently underestimate the effects of LBP on individual and public health. Most of these investigations have limited follow-up periods of only 2–3 years, potentially overlooking the longer-term patterns and implications of LBP recurrence [16,18,21]. Consequently, these limitations highlight the need for extended observation periods to fully understand the progression of the condition and its broader implications.

Several methodological challenges have restricted previous studies to shorter observation periods. One significant obstacle is the historical lack of national-level data collection systems, which has made large-scale cohort studies difficult to conduct. Consequently, researchers have often relied on smaller, single-institution studies with follow-up periods typically limited to 1–3 years [16,18,21]. These limitations in study duration and sample size have hindered a comprehensive understanding of long-term LBP patterns. Furthermore, the most significant barrier to progress in LBP research is the inconsistency in how LBP is defined and diagnosed across studies, resulting in varied findings and challenges in comparing results [22,23]. The diverse manifestations of LBP, ranging from self-reported discomfort to clinically documented disability, have further contributed to these discrepancies. This lack of standardization has significantly hindered the ability to conduct large-scale, population-based studies that could provide more definitive insights into the patterns and progression of the condition [24]. Such inconsistency has also limited the comparability of findings across studies and prevented the development of consistent epidemiological insights. By utilizing the NHIS database, which includes standardized diagnostic coding, uniform data collection protocols, and comprehensive national coverage, our study aims to overcome these limitations and enable a more reliable characterization of long-term LBP patterns in a large population. Furthermore, examining the influence of socioeconomic and demographic factors on LBP recurrence is essential to understanding disparities in disease burden and healthcare utilization. Identifying vulnerable populations with higher recurrence risks or limited access to care can inform more equitable clinical decision-making and public health policy.

The National Health Insurance Service (NHIS) database in South Korea provides a unique opportunity to overcome many limitations of previous studies. This extensive database includes comprehensive health records, patient demographics, and data on healthcare utilization for the entire Korean population. The broad scope and long-term structure of the NHIS database provides a valuable resource for examining long-term trends in LBP, which is characterized by its fluctuating nature and varying manifestations. The nationwide coverage and standardized methods of data collection further enhance its utility for conducting population-based research that surpasses the constraints of conventional studies.

This study analyzes a nationwide cohort of patients with low back pain (LBP) over a 17-year period using data from the National Health Insurance Service (NHIS) of Korea. The study seeks to profile a newly established nationwide cohort and describes sociodemographic characteristics to gain deeper insights into the course, progression, and recurrence patterns of LBP in a large-scale population. Therefore, this study aims to characterize the recurrence patterns and healthcare utilization of low back pain over a 17-year period using a nationwide population-based cohort. These findings are expected to enhance understanding of the long-term burden of LBP and provide an epidemiological foundation that can inform future research on clinical practice and public health strategies.

## 2. Materials and Methods

### 2.1. Data Sources

The National Health Information Database (NHID) of South Korea, established in 2002, integrates extensive healthcare data from the NHISS. This nationwide database covers the entire population of South Korea of approximately 50 million people, and it is organized into five distinct components. For this study, we employed four of these components, excluding the long-term care insurance database. The first component contains demographic data, including age, sex, residence, health insurance premium level, disability classification, and mortality status of individuals. The second component includes health examination data, which comprises lifestyle factors (e.g., smoking habits, alcohol consumption, and physical activity patterns) and clinical measurements (e.g., anthropometric data, blood pressure readings, and vision tests). The third component tracks healthcare utilization, recording diagnoses, healthcare visit dates, hospitalization duration, treatment modalities, medical expenses, and healthcare facility identifiers. The fourth component provides detailed information about healthcare facilities, including their classification, geographic location, and institutional capacity (measured by bed count and physician numbers). This research protocol was approved by the Institutional Review Board of University (IRB No. U-10-09-001, 14 August 2020).

### 2.2. Cohort Description

Population data from the NHISS were used in this study to establish the study cohort. The initial cohort included individuals aged 18 and 65 years as of 2010, totaling 35,194,498 people. Their medical records were analyzed over 17 years, from 1 January 2002 to 31 December 2018. Among this cohort, we identified patients who sought medical care for back pain (ICD-10 code M54.5) at least once in 2010. Patients with specific pathologies that could lead to low back pain, such as intervertebral disc disorders (M51), spinal stenosis (M48.0), and lumbar radiculopathy (M54.1), were excluded as these conditions have distinct clinical courses and management approaches. While some individuals may have initially received an M54.5 code and later transitioned to more specific diagnoses (e.g., M47.8, M51.1) during the study period, only episodes coded as M54.5 were included in our analysis. As such, patients who did not receive subsequent M54.5 diagnoses were not considered to have experienced a recurrence. Additionally, we excluded individuals who had undergone a disability rating assessment at any point during the study period. The disability rating system in South Korea identifies people with severe functional limitations who typically exhibit different healthcare utilization patterns compared to the general population. By excluding these cases, we focused on capturing the natural history of non-specific LBP in the general working-age population, ensuring greater homogeneity in our study cohort. The final study cohort comprised 3,086,665 patients whose complete medical records from the 6179-day study period (2002–2018) were reviewed. We specifically extracted healthcare visits related to back pain to investigate the cyclical patterns and recurrence characteristics of the condition.

### 2.3. Income-Based Variables

From the eligibility database, we analyzed two key socioeconomic indicators: health insurance premium levels and the Regional Health Vulnerability Index (RHVI). Health insurance premium levels are divided into 10 levels based on national standards, where level 1 represents the lowest income group and level 10 represents the highest income group. This premium-based stratification reflects the income status of all citizens in ascending order.

The RHVI was used to assess regional disparities in the healthcare system across different residential areas. It takes into account various factors, including the distribution of healthcare resources, healthcare needs, socioeconomic status of residents, and local governmental financial capacity. The RHVI is calculated based on residential area codes, with level 1 indicating regions with the best healthcare accessibility and level 10 representing regions with the most significant barriers to healthcare access. Among the study population, 597 individuals had missing RHVI data.

### 2.4. Healthcare Utilization Variables

To analyze the patterns of LBP recurrence and associated healthcare utilization, we examined several key variables derived from the 17-year patient records. In the NHIS database, each row of data represents a single healthcare visit for an individual patient. Using these visit-level data, we constructed episode-based variables to characterize the recurrent nature of LBP.

The duration of each episode was calculated as the period between the first and last healthcare visits within an episode, measured in days. This metric captures the clinical duration of an LBP episode requiring medical attention. The total duration of all episodes was determined by summing the durations of all episodes experienced by a patient over the study period, providing a cumulative measure of time spent with symptomatic LBP requiring care.

The number of healthcare visits per episode was counted as the total visits occurring within each defined episode. This variable reflects the intensity of healthcare utilization within a single episode, encompassing both outpatient visits and inpatient admissions. Patients with the same episode duration might have different numbers of healthcare visits, indicating varying intensity of care needed.

These variables allowed us to comprehensively examine both the temporal patterns of LBP recurrence and the associated healthcare utilization burden across the study population. All metrics were analyzed both at the patient level and at the episode level to examine progression patterns across sequential episodes.

### 2.5. Data Analysis

Statistical analyses were performed using Stata version 17.0 (StataCorp, College Station, TX, USA) to evaluate patient characteristics and patterns of back pain. Patient demographics, including age, sex, and socioeconomic indicators, were analyzed. Owing to privacy protection regulations, birth month and day were withheld; therefore, patient age was calculated by subtracting the birth year from the hospitalization year.

To analyze back pain patterns and their associated burden, we examined both the frequency and duration of back pain episodes. An episode was defined as a series of healthcare visits separated by intervals of ≤30 days between consecutive visits. A new episode was considered to begin when more than 30 days elapsed since the last visit of the previous episode. This 30-day criterion was based on established methodology from the previous literature [25]. We examined the age at the first back pain-related healthcare visit and counted the total number of episodes recorded during the study period. The duration of each episode was calculated as the period between the first and last healthcare visits within that episode. The total duration of back pain was determined by summing the durations of all episodes over the follow-up period. Additionally, we recorded the number of healthcare visits per episode and calculated the overall follow-up duration for each patient.

## 3. Results

### 3.1. Characteristics of the Patients with Back Pain

This study included 3,086,665 patients who experienced at least one episode of LBP, as identified using healthcare visits in 2010 (Table 1). The estimated prevalence of LBP within this cohort was 8.8%. The demographic profile revealed a mean patient age of 44.6 ± 12.1 years, with females accounting for 56.2% of the cohort (Table 1). The mean age at the onset of the first episode was 43.4 ± 12.1 years. An analysis of health insurance premium levels showed that 43.9% of patients were categorized within levels 1 to 5, reflecting a slightly lower representation of higher-income individuals in the cohort. The mean RHVI score for this population was 46.7 ± 5.5, which is below the national average of 50, indicating that most patients resided in regions with relatively better healthcare accessibility.

Table 2 details the relationship between RHVI and health insurance premium levels. Regions with the lowest RHVI score (level 1) exhibited a significant concentration of higher-income residents, with 33% of patients in these regions falling within the top three premium levels (8–10). Conversely, regions with the highest RHVI scores (level 10) displayed a relatively uniform distribution across premium levels, with approximately 10% of patients in each decile. However, these areas showed a significantly lower representation (6.6%) in the highest premium decile (level 10), suggesting that fewer high-income residents live in these vulnerable regions.

### 3.2. Recurrence of Back Pain

An analysis of patient data over the 6179-day study period (2002–2018) revealed distinct patterns in the recurrence of LBP as summarized in Table 3. This table presents patient-level statistics, showing how individual patients experienced LBP episodes throughout the study period, including the frequency of episodes, their duration, and associated healthcare utilization. Patients experienced an average of 3.5 ± 9.6 episodes during this period. Most patients (79.4%, *n* = 2,451,950) experienced more than one episode throughout the study period. Examining the progression from the first to the tenth episode, more than 73% of patients at each stage went on to experience subsequent episodes (Table 4). To understand how the characteristics of LBP changed with recurrence, Table 4 tracks the progression of episode characteristics from the first to the tenth occurrence. Unlike Table 3, which presents overall patient experiences, this episode-level analysis reveals how duration, healthcare utilization, and intervals between episodes evolved over time, which is critical for understanding the dynamic nature of recurrent LBP. Among patients who experienced their first episode during the study period, 77.1% (*n* = 1,890,871) went on to have at least one additional episode.

Each episode of LBP lasted an average of 15.3 ± 46.5 days, with the cumulative duration of symptoms across all episodes averaging 67.5 ± 172.4 days. Patients with multiple episodes experienced significantly longer total symptom durations compared to those with only a single episode. The average time span between the first and last recorded episodes was 3266.4 ± 286.5 days.

### 3.3. Burden of Multiple Episodes of Back Pain

An analysis of LBP episodes (Table 4) revealed that later episodes tended to last longer than earlier ones. The average duration of the first episode was 7.6 ± 28.1 days, which progressively increased to 11.4 ± 35.8 days by the third episode, 15.2 ± 43.7 days by the sixth episode, and 19.5 ± 51.3 days by the tenth episode. A similar pattern was observed in healthcare utilization, with patients attending an average of 2.8 ± 6.8 visits during their first episode, rising to 3.9 ± 10.3 visits by the tenth episode.

**Figure 1:**
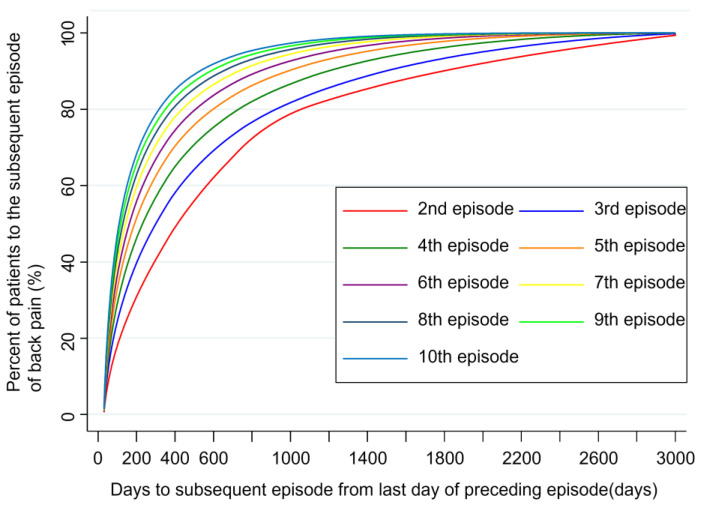
Patients (%) by number of days to the subsequent episode. Figure 1 illustrates the decreasing time intervals between successive episodes of back pain in patients. It shows a trend where patients with more episodes experience shorter gaps between episodes. For example, the figure highlights that 50% of patients had their second episode around 700 days after the first, but this interval reduces significantly with each subsequent episode, demonstrating quicker recurrence of back pain in frequent sufferers.

The interval between LBP episodes decreased as patients experienced more episodes (Figure 1). Patients with a higher number of episodes, such as those with nine episodes, experienced subsequent episodes more quickly than those with fewer episodes. For example, after the first episode, approximately half of the patients had their second episode around 500 days later. This interval progressively shortened with each subsequent episode: approximately half of the patients had their third episode approximately 300 days after the second, their fourth episode approximately 240 days after the third, and their fifth episode approximately 200 days after the fourth.

The analyses presented in Table 3 and Table 4, along with Figure 1, provide different but interconnected perspectives on LBP recurrence. While Table 3 characterizes the overall burden at the patient level across the entire study period, Table 4 and Figure 1 demonstrate how the nature of LBP episodes changes with each recurrence, highlighting both the progressive lengthening of episodes and the shortening of pain-free intervals over time.

## 4. Discussion

The nationwide cohort study, spanning 17 years, represents the first comprehensive longitudinal analysis of the LBP incidence, patterns, progression, and disease burden based on the NHIS database of South Korea. On average, patients experienced 5.0 ± 4.9 episodes of LBP during the study period. The findings highlight that LBP is not merely an isolated condition but a recurrent issue that poses significant challenges to patients and healthcare systems. The study revealed distinct patterns in the frequency, duration, and healthcare utilization associated with LBP episodes. Furthermore, as the number of episodes increased, the intervals between episodes shortened, while healthcare utilization intensified, reflecting a progressive burden on healthcare resources.

Demographic analyses revealed that LBP was most prevalent among individuals aged 40–59 years and more commonly observed in women, aligning with global epidemiological trends [1,26]. Furthermore, demographic characteristics and regional health vulnerabilities, which act as determinants and triggers of healthcare utilization, indicate that weaker regional health systems lead to higher doctor visits compared to more populated and affluent areas. These findings are crucial in establishing a common foundation for demographic, social, and clinical research, as highlighted in the third year of the LBP Congress.

Socioeconomic indicators analyzed in this study include the health insurance premium deciles and RHVI, and this analysis was used to explore their relationship with LBP (Table 2). The analysis revealed that 34.9% of patients with LBP were in the top three income deciles, compared to 26.8% in the bottom three deciles. This disparity suggests that individuals with higher socioeconomic status (SES) may report and seek treatment for LBP more often, potentially owing to better access to healthcare services. The RHVI analysis indicated an even assigning of patients with LBP between vulnerable and less vulnerable regions. However, higher-income patients were more commonly located in less vulnerable regions, highlighting potential disparities in healthcare access. Conversely, income distributions were similar among individuals in more vulnerable regions, which may reflect the underreporting of LBP or limited access to medical care. These observations highlight that socioeconomic factors may be relevant considerations in the context of LBP healthcare delivery. The observed demographic distribution suggests that factors such as healthcare accessibility and treatment-seeking behavior could vary across different socioeconomic groups. Healthcare providers may benefit from considering these potential socioeconomic contexts when developing treatment plans for patients with LBP, although further research would be needed to establish direct relationships between these factors and clinical outcomes.

The findings of this study revealed a high prevalence of recurrent LBP episodes, with 79.4% of patients experiencing at least one recurrence. Successive episodes were characterized by progressively shorter intervals between episodes and longer durations, increasing from an average of 7.6 days in the first episode to 19.5 days in the tenth episode. Additionally, the frequency of healthcare visits per episode increased over time, rising from a mean of 2.8 (±6.8) visits during the first episode to 3.9 (±10.3) visits by the tenth episode. These patterns emphasize the cumulative burden LBP places on patients and healthcare systems. The findings reaffirm that recurrent back pain is a common and episodic condition [27,28], imposing a progressively greater burden on affected individuals and the healthcare infrastructure over time. Our findings, particularly the progressive shortening of intervals and increasing episode duration, are consistent with prior studies reporting the cyclic and worsening nature of LBP over time [15,18]. These studies similarly observed that patients with recurrent LBP tend to experience more frequent and prolonged episodes, highlighting the need for early and sustained intervention strategies. The frequent recurrence of healthcare visits for LBP may, in part, be attributed to limitations in diagnostic precision, which often result in the use of symptomatic treatments such as analgesics rather than interventions targeting the underlying cause. This clinical approach may lead to only temporary symptom relief and contribute to repeated medical consultations, as suggested in previous studies [22,23].

The key contribution of this study lies in highlighting the dynamic recurrence patterns of LBP (Table 4 and Figure 1). With an increasing number of episodes, the intervals between episodes became progressively shorter while the duration of each episode lengthened. These trends indicate that LBP imposes a growing burden on both affected individuals and healthcare systems. These findings align with that of previous research suggesting that LBP is a cyclic condition that evolves over time [16,29]. The 17-year follow-up in this study represents a significant methodological enhancement compared to that of previous studies, which primarily examined shorter timeframes and may have underestimated the complexity and long-term effects of LBP [16,17,21]. By adopting a longitudinal approach, this study underscores the need for healthcare systems to shift from episodic management to comprehensive, longer-term strategies for addressing LBP.

The progression patterns observed in LBP closely mirror findings from our previous research on neck pain [16,17,18,19,20,21,30]. In that study, we identified similar trends among patients with recurrent neck pain, characterized by (i) prolonged episode duration, (ii) increased frequency of healthcare visits, and (iii) shorter intervals between episodes (Table 4 and Figure 1). This consistency across LBP and neck pain suggests a shared trajectory in chronic musculoskeletal conditions, where patients with multiple episodes tend to experience more frequent and prolonged symptoms, requiring increasingly intensive healthcare utilization. Similar patterns observed in both conditions are further supported by recent research [25], which highlights persistent deficits in neuromotor and sensorimotor control, even during pain-free intervals. Although our data do not directly evaluate these functional impairments, the consistent findings across our studies on LBP emphasize the critical importance of early intervention strategies to mitigate the progression and recurrence of chronic musculoskeletal conditions [31]. Such interventions might include evidence-based approaches like targeted exercise programs, patient education, and multidisciplinary rehabilitation, which could be particularly effective when implemented before the pattern of shortening intervals between episodes becomes established. In the context of the recurrent nature of LBP observed in this study, it may be meaningful to consider commonly used intervention strategies that have been recommended in clinical practice. Although our study did not directly evaluate treatment modalities, future studies could explore how these interventions may influence long-term recurrence patterns or healthcare utilization trends.

This study has some limitations. First, it relies on a single year of data, which limits its ability to capture the long-term patterns and fluctuations associated with LBP. Furthermore, as a chronic condition, LBP often exhibits variations over time, and the absence of multi-year data overlooks critical insights into its recurrence and associated healthcare utilization. Second, the lack of a control group prevents direct comparisons with individuals who did not experience LBP, thereby limiting the ability to accurately assess the relative impact of LBP on healthcare resource utilization and work-related outcomes over time. This limitation makes it challenging to validate the findings and assess whether the observed patterns are truly unique to LBP or could be influenced by other factors. Third, the absence of statistical modeling restricts the ability to determine causal relationships between variables. Such modeling would be essential to understand how changes in one factor—such as episode frequency—may lead to the observed outcomes. Furthermore, the lack of data on treatment modalities limits our ability to evaluate how specific interventions, such as medication, physical therapy, or surgical procedures, may have influenced the progression or recurrence of LBP over time. Additionally, excluding individuals who do not seek medical care may result in an underestimation of the true prevalence of LBP, as this population is likely to include those who manage their symptoms independently without seeking formal treatment. This methodological limitation is inherent to studies using healthcare administrative data, creating a systematic difference between clinically observed prevalence and community prevalence. Lastly, although this study provides meaningful longitudinal insights into the recurrence of LBP, it does not incorporate predictive modeling, which limits its ability to directly identify risk factors for LBP recurrence. Future studies are warranted to address this gap by integrating therapeutic data and causal inference frameworks.

## 5. Conclusions

This 17-year nationwide cohort study underscores the dynamic and progressive nature of LBP, with patients experiencing an average of 5.0 episodes characterized by progressively shorter intervals between recurrences and longer episode durations over time. LBP was most prevalent among middle-aged adults (40–59 years) and women. SES and regional health vulnerability were significant factors influencing recurrence rates and healthcare utilization patterns. Higher-income individuals were more likely to report LBP and seek treatment, while patients in vulnerable regions faced disparities in access to care. By analyzing the recurrence patterns and longitudinal course of LBP in a large-scale population, this study fulfills its objective of providing deeper insights into the natural history of the condition. Furthermore, the findings offer meaningful evidence to inform the development of long-term clinical strategies and public health interventions aimed at mitigating the cumulative burden of recurrent LBP. These results highlight the importance of shifting healthcare systems from episodic care models to comprehensive, sustained management approaches for chronic musculoskeletal conditions.

## Figures and Tables

**Table 1:** Characteristics of patients who had healthcare visits owing to back pain in 2010.

Characteristics		*n* = 3,086,665
*Patient age in 2010 (n, %)*		
<30 years	419,809	(13.6%)
30–39 years	650,289	(20.7%)
40–49 years	801,983	(26.0%)
50–59 years	848,458	(27.5%)
>59 years	376,126	(12.2%)
*Sex (n, %)*		
Male	1,353,551	(43.9%)
Female	1,733,114	(56.2%)
*Health insurance premium decile (n, %)*		
Level 1	333,510	(10.8%)
Level 2	205,642	(6.7%)
Level 3	255,974	(8.3%)
Level 4	262,461	(8.5%)
Level 5	298,599	(9.7%)
Level 6	319,567	(10.4%)
Level 7	332,505	(10.8%)
Level 8	346,008	(11.2%)
Level 9	361,081	(11.7%)
Level 10	371,318	(12.0%)
*Regional Health Vulnerability Index ^a^ (n, %)*		
Level 1	276,097	(9.0%)
Level 2	305,137	(9.9%)
Level 3	299,732	(9.7%)
Level 4	356,010	(11.5%)
Level 5	312,286	(10.1%)
Level 6	265,137	(8.6%)
Level 7	323,910	(10.5%)
Level 8	273,606	(8.8%)
Level 9	338,373	(11.0%)
Level 10	335,780	(10.9%)

^a^ Data for the specific variable were available for only 3,086,068 patients. Percentages may not sum to exactly 100% due to rounding to one decimal place.

**Table 2:** Distribution of health insurance premium deciles across regional health vulnerability index levels in 2010 (*n* = 3,086,068).

	Health Insurance Premium Decile (n, %)										
	Level 1	Level 2	Level 3	Level 4	Level 5	Level 6	Level 7	Level 8	Level 9	Level 10	Total
**Level 1**	22,953 (6.9%)	14,041 (6.8%)	21,558 (8.4%)	22,517 (8.6%)	23,933 (8.0%)	25,173 (7.9%)	26,431 (8.0%)	28,925 (8.4%)	35,167 (9.7%)	55,399 (14.9%)	276,097 (8.9%)
**Level 2**	26,491 (8.0%)	17,583 (8.6%)	24,768 (9.7%)	25,263 (9.6%)	27,993 (9.4%)	29,780 (9.3%)	32,038 (9.6%)	34,816 (10.1%)	38,780 (10.7%)	47,625 (12.8%)	305,137 (9.9%)
**Level 3**	28,093 (8.4%)	18,950 (9.2%)	24,204 (9.5%)	23,713 (9.0%)	26,680 (8.9%)	28,656 (9.0%)	30,913 (9.3%)	34,155 (9.9%)	38,898 (10.8%)	45,470 (12.2%)	299,732 (9.7%)
**Level 4**	35,112 (10.5%)	23,477 (11.4%)	31,107 (12.2%)	31,169 (11.9%)	35,338 (11.8%)	37,129 (11.6%)	38,462 (11.6%)	41,015 (11.8%)	42,528 (11.8%)	40,673 (10.9%)	356,010 (11.5%)
**Level 5**	34,083 (10.2%)	21,590 (10.5%)	26,024 (10.2%)	26,026 (9.9%)	29,795 (10.0%)	32,297 (10.1%)	34,119 (10.3%)	35,515 (10.3%)	36,687 (10.2%)	36,150 (9.7%)	312,286 (10.1%)
**Level 6**	28,978 (8.7%)	18,008 (8.8%)	22,930 (9.0%)	22,708 (8.7%)	25,768 (8.6%)	27,458 (8.6%)	28,883 (8.7%)	30,889 (8.9%)	31,098 (8.6%)	28,417 (7.7%)	265,137 (8.6%)
**Level 7**	36,773 (11.0%)	23,671 (11.5%)	26,241 (10.2%)	27,189 (10.4%)	30,672 (10.3%)	34,350 (10.8%)	35,893 (10.8%)	36,164 (10.4%)	36,880 (10.2%)	36,077 (9.7%)	323,910 (10.5%)
**Level 8**	34,382 (10.3%)	19,920 (9.7%)	22,048 (8.6%)	23,206 (8.8%)	27,096 (9.1%)	29,329 (9.2%)	30,650 (9.2%)	30,166 (8.7%)	29,109 (8.1%)	27,700 (7.5%)	273,606 (8.9%)
**Level 9**	42,614 (12.8%)	25,122 (12.2%)	29,479 (11.5%)	30,679 (11.7%)	35,307 (11.8%)	37,325 (11.7%)	36,753 (11.1%)	36,287 (10.5%)	35,820 (9.9%)	28,987 (7.8%)	338,373 (11.0%)
**Level 10**	43,943 (13.2%)	23,123 (11.2%)	27,531 (10.8%)	29,932 (11.4%)	35,975 (12.1%)	37,947 (11.9%)	38,341 (11.5%)	38,065 (11.0%)	36,112 (10.0%)	24,811 (6.7%)	335,780 (10.9%)
**Total**	333,422 (10.8%)	205,485 (6.76%)	255,890 (8.3%)	262,402 (8.5%)	298,557 (9.7%)	319,444 (10.4%)	332,483 (10.8%)	345,997 (11.2%)	361,079 (11.7%)	371,309 (12.0%)	3,086,068 (100%)

**Table 3:** Frequency, duration, and healthcare visits of back pain episodes over 6179 days (*n* = 3,086,665).

Characteristics	Values	% or SD
*Onset age at first episode (n, %)*		
<30 years	435,196	(14.1%)
30–39 years	649,714	(21.0%)
40–49 years	823,188	(26.7%)
50–59 years	842,107	(27.3%)
>59 years	336,460	(10.9%)
*Number of episodes during the study period (mean, SD)*	5.0	4.9
*Number of patients with (n, %)*		
A single episode	634,715	(20.6%)
Two episodes	561,079	(18.2%)
Three episodes	420,040	(13.6%)
Four episodes	310,054	(10.0%)
Five episodes	231,341	(7.5%)
Six episodes	176,798	(5.7%)
Seven episodes	137,288	(4.5%)
Eight episodes	108,058	(3.5%)
Nine episodes	86,637	(2.8%)
Ten episodes	69,744	(2.3%)
More than ten episodes	359,911	(11.4%)
*Mean duration of each episode in days (mean, SD)*	15.3	46.5
Patients with a single episode (*n* = 634,715)	6.5	23.2
Patients with two or more episodes (*n* = 2,451,950)	13.31	40.2
*Total duration of all episodes in days (mean, SD)*	67.5	172.4
Patients with a single episode (*n* = 634,715)	8.6	24.1
Patients with two or more episodes (*n* = 2,451,950)	107.7	253.4
*Follow-up duration per patient (mean, SD)*	3266.4	286.5
*Mean number of healthcare visits per episode (mean, SD)*	3.5	9.6

**Table 4:** Characteristics of back pain cycle from the first to tenth episode.

	Episodes ^a^									
	1st	2nd	3rd	4th	5th	6th	7th	8th	9th	10th
**Total number of patient** **s at each episode (n)**	3,086,665	2,451,950	1,890,871	1,470,831	1,160,777	929,436	752,638	615,350	507,292	420,655
**Recurrence rate of a new back pain episode relative to the previous episode (%)**	- ^b^	79.4	77.1	77.8	78.9	80.1	81.0	81.8	82.4	82.9
**Duration of each episode in days** **(** **mean, (** **SD** **)** **)**	7.6 (28.1)	10.0 (32.5)	11.4 (35.8)	12.8 (38.8)	14.1 (41.2)	15.2 (43.7)	16.4 (46.7)	17.4 (48.2)	18.5 (50.6)	19.5 (51.3)
**Number of healthcare visits at each episode (mean, (SD))**	2.8 (6.8)	3.0 (7.00)	3.2 (7.9)	3.3 (8.3)	3.4 (8.6)	3.5 (8.4)	3.6 (9.0)	3.7 (8.8)	3.8 (9.4)	3.9 (10.3)

^a^ This table shows data from the first to tenth episode, accounting for 89% of total episodes. ^b^ Data are unavailable owing to the absence of a prior episode for the first episode.

## Data Availability

The data supporting this study cannot be publicly shared owing to participant privacy concerns and regulations established by the Ministry of Health and Welfare, South Korea.
